# Initial Report on Differential Expression of Sprouty Proteins 1 and 2 in Human Epithelial Ovarian Cancer Cell Lines

**DOI:** 10.1155/2012/373826

**Published:** 2012-11-29

**Authors:** Samar Masoumi Moghaddam, Afshin Amini, Ai-Qun Wei, Mohammad Hossein Pourgholami, David Lawson Morris

**Affiliations:** ^1^Cancer Research Laboratories, Department of Surgery, St. George Hospital, The University of New South Wales, Sydney, NSW 2217, Australia; ^2^Department of Orthopedic Surgery, St. George Hospital, The University of New South Wales, Sydney, NSW 2217, Australia; ^3^Department of Surgery, St. George Hospital, The University of New South Wales, Sydney, NSW 2217, Australia

## Abstract

Sprouty (Spry) proteins, modulators of receptor tyrosine kinase signaling pathways, have been shown to be deregulated in a variety of pathological conditions including cancer. In the present study we investigated the expression of Spry1 and Spry2 isoforms in a panel of human ovarian cancer cell lines in vitro. Our western blot analysis showed nonuniform patterns of Spry expression in the cancer cells, none of which conformed to the pattern observed in the normal ovarian epithelial cells employed as the control. Among the seven cancer cell lines studied, Spry1 was expressed lower in four cell lines and higher in one as compared with the control. As for Spry2, four cell lines showed lower and two exhibited higher expression. Results from RT-PCR assay raised the possibility that Spry protein levels may not necessarily correspond with its expression at mRNA level. Our immunostaining study revealed that Spry2 was predominantly distributed within the whole cytoplasm in vesicular structures whereas Spry1 was found in both the cytoplasm and nucleus. This might provide clues to further investigation of Spry mode of action and/or function. Collectively, our study unveiled the differential expression of Spry1 and Spry2 proteins in various ovarian cancer cell lines.

## 1. Introduction

Aberrant activity of various receptor tyrosine kinases (RTKs) plays an essential role in the pathogenesis of malignancies. Sprouty (Spry) proteins represent a major class of ligand-inducible inhibitors of RTK-dependent signaling pathways. dSpry (*Drosophila melanogaster* Spry) was initially defined by Hacohen et al. in 1998 as an inhibitor of fibroblast growth factor (FGF)-mediated tracheal branching during *D. melanogaster* development [1]. They also identified three human homologs of dSpry, designated Spry1–3, in a search of the Expressed Sequence Tag (EST) database. The fourth mammalian homolog was later discovered in mice [2] and humans [3]. Spry proteins, in particular Spry1, Spry2, and Spry4 isoforms that may have an important role in controlling growth signals, are evidently deregulated in some pathological conditions including cancer. Several studies have reported Spry up- or downregulation in a variety of neoplasms including breast [4], prostate [5–7], hepatocellular [8, 9], colon [10], and lung cancer [11, 12], as well as melanoma [13, 14] and gastrointestinal stromal tumors [15], implicating Spry as a possible tumor suppressor that could be potentially employed as a tumor marker and an interesting target for drug intervention. On this basis, we intended to investigate the role of Spry in ovarian cancer, the seventh leading cancer in women and the second cause of death from gynaecological malignancies worldwide [16].

As an initial attempt, we aimed in this study to evaluate the expression pattern of Spry1 and Spry2 isoforms in a panel of human epithelial ovarian cancer cell lines. Since alteration in Spry expression in this pathological condition was anticipated, primary human ovarian epithelial cells were also employed as the control, against which the expression pattern in the cancer cells could be compared.

## 2. Materials and Methods

### 2.1. Cell Culture

Human ovarian cancer cell lines OVCAR-3, SKOV-3, and 1A9 were obtained from the American Type Culture Collection (ATCC) (Manassas, VA, USA). Other human ovarian cancer cells, CAOV-3, A2780, OV-90, and IGROV-1, were a kind gift from Dr. Yong Lee (Department of Radiology, St George Hospital, The University of New South Wales, Australia). The primary human ovarian surface epithelial cell line HOSEpiC was obtained from SienCell (SienCell, CA, USA). All cell lines were maintained in a humidified 5% CO_2_ incubator at 37°C in their respective medium as follows: OVCAR-3, SKOV-3, 1A9, A2780, and IGROV-1 cells in RPMI-1640 (Invitrogen, CA, USA), CAOV-3 in DMEM (Invitrogen, CA, USA), OV-90 cells in a 1 : 1 mixture of MCDB 105/Medium 199 (Sigma-Aldrich, Missouri, USA), and HOSEpiC in OEpiCM (SienCell, CA, USA). The culture media used were all supplemented with 10% fetal bovine serum and 1% penicillin-streptomycin mixture (Invitrogen, CA, USA).

### 2.2. Immunocytochemistry

OVCAR-3, SKOV-3, and HOSEpiC cells were seeded onto sterile glass coverslips in a 6-well tissue culture plate at an initial density of 2.5 × 10^5^ cells/well and maintained in RPMI medium supplemented with 10% fetal bovine serum at 37°C in a humidified, 5%  CO_2_ atmosphere. At 50% confluence, the cells were fixed in 0.1% sodium azide plus 0.5% formaldehyde in phosphate-buffered saline (PBS) (Sigma-Aldrich, Missouri, USA) for one hour at room temperature. This was followed by one hour incubation with 70% ethanol/PBS at 4°C for permeabilization and further fixation. In order to block nonspecific binding of the antibodies, the coverslips were immersed in 1% bovine serum albumin (BSA) in PBS for one hour at room temperature. Cells were then incubated overnight at 4°C with monoclonal anti-Spry1 and anti-Spry2 antibodies (1 : 20 in 1% BSA/1x PBS) (Abnova, Taiwan), with the exception of the negative control samples to which no primary antibody was applied. Incubation with the secondary antibody was subsequently applied to all samples using Alexa Flour 488 chicken anti-mouse IgG (1 : 500 in 1% BSA/1x PBS) (Invitrogen, CA, USA) for 1 hour at 4°C in dark. Next, the cells were counter-stained with propidium iodide (1 : 500) for 3 minutes and the coverslips were mounted with gelatine glycerol and stored at 4°C in dark. Each step was followed by rinsing with PBS. The cells were visualized by laser scanning confocal microscope (Olympus, USA) and X60 oil immersion lense. The FluoView software (version 4.3, Center Valley, PA) was used to overlay the images.

### 2.3. Western Blotting

Cells were homogenized in a protein lysis buffer (RIPA buffer) containing 10% protease inhibitor (Sigma-Aldrich, Missouri, USA) and the protein concentrations were quantified by BioRad protein assay (Bio-Rad, CA, USA). Then, the same amounts of the proteins were separated by sodium dodecyl sulfate-polyacrylamide gel electrophoresis, transferred to PVDF membranes (Millipore Corporation, MA, USA), and incubated overnight at 4°C with a 1 : 1000 dilution of anti-Spry1 or anti-Spry2 mouse monoclonal antibodies (Abnova, Taiwan). Membranes were washed and treated with goat anti-mouse secondary antibody conjugated to horseradish peroxidase (1 : 5000 dilution; Santa Cruz Biotechnology, CA, USA) for 1 hour at room temperature. Similar process was carried out for GAPDH protein with a 1 : 20000 dilution of anti-GAPDH mouse monoclonal antibody (Sigma-Aldrich, Missouri, USA). Using the ImageQuant LAS 4000 Biomolecular imager and ImageQuant software (GE Healthcare, UK), the antigen-antibody reaction was digitized, band densitometry was quantified and the data was normalized against the values of GAPDH protein expression. Quantitative analysis of the protein expression was otherwise performed through normalizing the data from cancer cell lines against those from HOSEpiC cells as the normal control, where the values were expressed in arbitrary units as a percentage of the protein expression in each cell line to that in HOSEpiC.

### 2.4. RT-PCR

Total RNA was isolated from seven ovarian cancer cell lines as well as the primary human ovarian surface epithelial cell line HOSEpiC in an RNase-free environment using RNeasy Plus Mini Kit (QIAGEN, Germany) as per the manufacturer's instructions. Possible contaminating DNA from RNA preparations was digested by DNA-free DNase Treatment and Removal Reagents (Ambion, Life Technologies, USA). The extracted RNA yield and purity were then determined by measuring the absorbance at 230, 260, and 280 nm using a NanoDrop 2000 Spectrophotometer (Thermo Fisher Scientific, MA, USA) followed by the evaluation of RNA integrity through gel electrophoresis for determination of 28S/18S ribosomal RNA (rRNA) ratio. Reverse transcription was performed using SuperScript III One-Step RT-PCR System with Platinum Taq DNA Polymerase (Invitrogen, CA, USA) in a Veriti 96-Well Thermal Cyclers (Applied Biosystems, CA, USA) according to the manufacturer's protocol with RNAse inhibitor (QIAGEN, Germany) added. The PCR amplification was carried out as follows: a 30 minute incubation at 56°C for cDNA synthesis, a 3 minute hot start at 94°C followed by 35 cycles of denaturation at 94°C for 30 seconds, annealing at 56°C for 30 seconds, and extension at 72°C for 30 seconds with a final extension at 72°C for 5 minutes. Transcripts amplification of Spry1 and Spry2 employed the following oligonucleotide primers based on the published sequence [17] to cross exon/intron regions:

 5′-CTGCAGGGGAAGTGCAAGTGTGGAGAA-3′ (forward) and
 5′-AAGCTTAGTTCAGGAGGTACAACCCAC-3′ (reverse) for Spry1, and
 5′-GGATCCCATTCGCTCATCTGCCAGGAA-3′ (forward) and
 5′-AAGCTTTGCTGGGTGAGGGCGTCTCTG-3′ (reverse) for Spry2.

Oligonucleotide primers of *β*-actin, 5′-ATATCGCCGCGCTCGTCGTC-3′ (forward) and 5′-AGTGGTACGGCCAGAGGCGT-3′ (reverse), were designed by NCBI Primer-BLAST (http://www.ncbi.nlm.nih.gov/tools/primer-blast/) and used as a reference in the same amplification conditions. Minus RT control (−RT) and no template control (NTC) were also included in the experiment to verify absence of genomic DNA in the RNA preparations and possible contamination of kit components, respectively. 10 *μ*L aliquots of the PCR products were separated on 1.5% agarose gel containing a 1 : 10,000 dilution of 10,000× SYBR Safe DNA gel stain (Invitrogen, CA, USA) by electrophoresis at 80 V and then observed using Bio-Rad Gel Doc UV Transilluminator 2000 (Bio-Rad, CA, USA). 

### 2.5. Human Samples and Immunohistochemistry

Following approval by the appropriate institutional ethics committee (South Eastern Sydney Local Health District Human Research Ethics Committee, NSW, Australia) and in accordance with the relevant guidelines, paraffin-embedded tumor tissue sections from patients with clinically diagnosed primary epithelial ovarian cancer were obtained and immunohistochemically stained. In brief, after deparaffinization, sections were pretreated and antigen retrieval was performed. The incubation with primary antibody was then performed at 4°C overnight using anti-Spry1 or anti-Spry2 monoclonal antibodies (Abnova, Taiwan) according to the manufacturer's protocol. This was followed by incubation with secondary antibody (Santa Cruz Biotechnology, CA, USA) and counterstaining with hematoxylin. Positive controls recommended by the manufacturer were used. A negative control, with no primary antibody applied, was also prepared for each sample. The stained slides were then observed by Leica DMLB microscope (magnification ×40) and photographed using Leica DC200 digital imaging system (Leica Microsystems, Wetzlar, Germany).

### 2.6. Statistical Analysis

All data presented are representative of three independent experiments. Statistical analyses were performed using GraphPad InStat (GraphPad Prism 5, San Diego, California, USA). Student's *t*-test was applied for unpaired samples and *P*  values < 0.05 were considered significant.

## 3. Results

### 3.1. Epithelial Ovarian Cancer Cell Lines Expressed Different Levels of Spry1 and Spry2 Proteins

**Figure 1:**
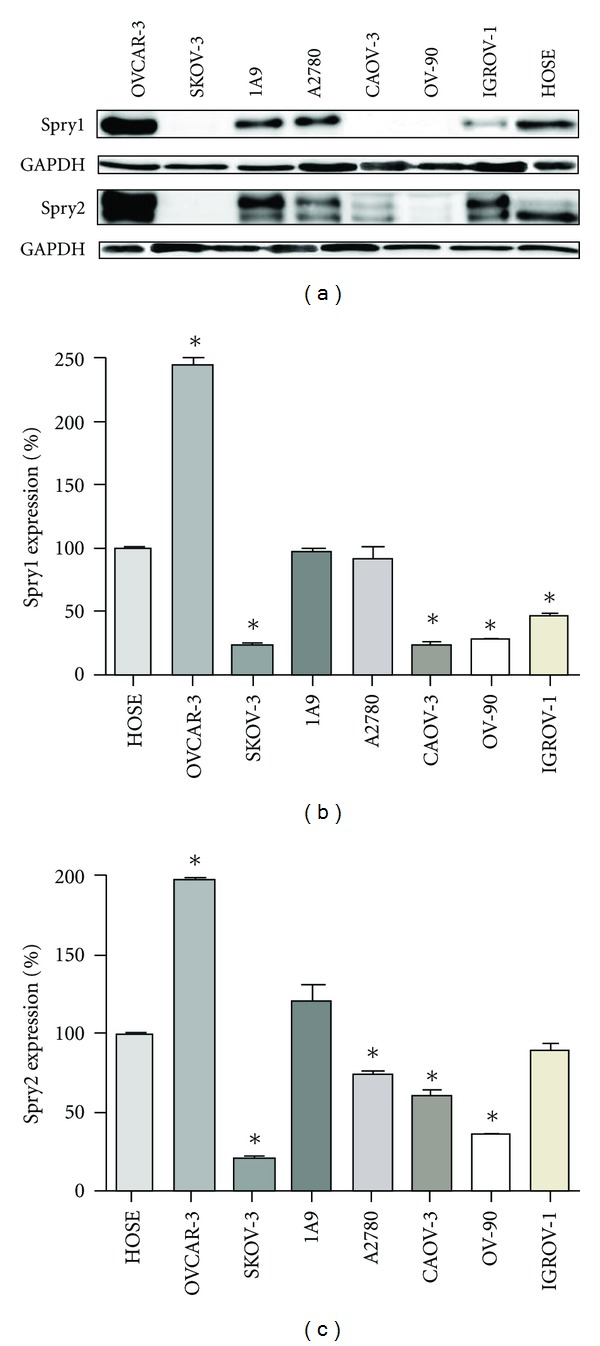
Expression of Spry1 and Spry2 proteins in a range of ovarian cancer cell lines compared with human ovarian surface epithelial cell line (HOSEpiC). (a) Western blot analysis of Spry1 and Spry2 expression in HOSEpiC, OVCAR-3, SKOV-3, 1A9, A2780, CAOV-3, OV-90, and IGROV-1 cell lines using GAPDH as a loading control. (b)-(c) Quantification of Spry expression in the cell lines yielded from at least three independent immunoblots similar to (a), using ImageQuant software. Loading amounts and quantification were normalized against GAPDH values. Moreover, the expression levels in cancer cell lines were normalized against those in HOSEpiC cells used as the normal control. The graphs represent the expression of Spry1 (b) and Spry2 (c) in arbitrary units after normalization. Altered expression of both isoforms was observed in the cancer cells as compared to the control. Data are shown as means ± SE. Significant values (*P* < 0.05) are indicated with asterisks.

Initial studies were carried out to investigate the expression of Spry1 and Spry2 proteins in seven commonly-used human epithelial ovarian cancer cell lines by western blot analysis using Spry1 and Spry2 specific antibodies. The epithelial ovarian cancer cells exhibited different levels of Spry expression (Figure 1(a)). While OVCAR-3 cells expressed high amount of Spry1 isoform, and 1A9 and A2780 cells showed a moderate level of expression, IGROV-1 cells expressed a low level of Spry1 and cell lines SKOV-3, CAOV-3, and OV-90 had almost no expression. As for Spry2, whereas OVCAR-3 and 1A9 exhibited high levels of expression and A2780 and IGROV-1 expressed it moderately, the expression levels were low for CAOV-3 and OV-90 cells and almost nil for SKOV-3. Of both isoforms, the highest and lowest levels of expression were seen with OVCAR-3 and SKOV-3 cells, respectively. In sum, our observation revealed nonuniform expression patterns of Spry1 and Spry2 across the seven cancer cell lines, with the expression levels ranging from almost nil to high. 

### 3.2. The Expression of Spry1 and Spry2 Proteins Was Altered in the Cancer Cell Lines Compared with Normal Ovarian Epithelial Cells

In order to investigate any possible alteration in the expression of human Spry1 and Spry2 proteins in ovarian cancer, western blotting was also performed on the protein lysates obtained from the primary human ovarian cells HOSEpiC (Figure 1(a)) and quantitative comparison of the protein expression was carried out using ImageQuant software (Figures 1(b)–1(c)). Our results indicated that Spry1 and Spry2 were moderately expressed in the normal cells. When the nonuniform expression patterns of Spry1 and Spry2 across the cancer cell lines were individually compared against this pattern, nonconformity was found. While the expression of Spry1 and Spry2 in OVCAR-3 was significantly higher (*P* values of 0.0012 and 0.0004, resp.), SKOV-3, CAOV-3, and OV-90 expressed significantly lower levels of Spry1 (*P*-values of 0.0002, 0.0015, and 0.0002, resp.) and Spry2 (*P*-values of <0.0001, 0.0146, and 0.0003, resp.), with SKOV-3 expressing the least. Although A2780 expression pattern of Spry1 was similar to that of the control group, this cell line expressed significantly higher levels of Spry2 (*P* value: 0.0032). IGROV-1 cells expressed Spry1 significantly lower (*P*-value: 0.0002) than the control group. Decline in IGROV-1 expression of Spry2, however, was not significant (*P*-value: 0.1074). 1A9 showed similar levels of Spry1 and insignificantly higher levels of Spry2 (*P*-value: 0.1657) as compared to the control. Amongst the cancer cell lines, OVCAR-3 and SKOV-3 expressed the highest and lowest levels of both isoforms, respectively. 

Taken together, while both Spry1 and Spry2 were moderately expressed in the normal ovarian cells employed as the control, alterations in the expression of Spry1 and/or Spry2 were found across all cancer cells studied. Spry1 was expressed lower in four cell lines and higher in one. As for Spry2, four cell lines showed lower and two exhibited higher expression, although increase in 1A9 expression of Spry2 was not significant.

### 3.3. Spry1 and Spry2 mRNAs Were Differentially Presented in Epithelial Ovarian Cancer Cells

**Figure 2:**
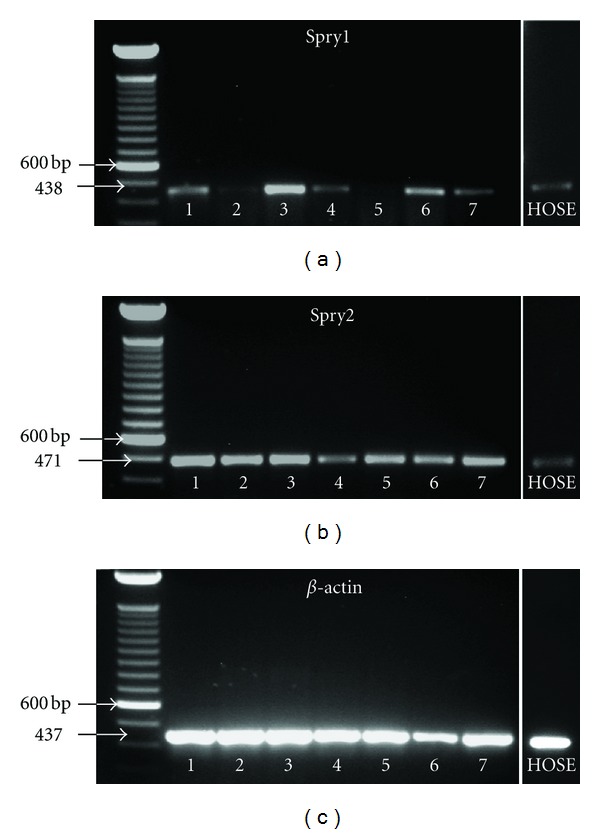
The presence of Spry1 and 2 mRNAs evaluated by RT-PCR in OVCAR-3 (1), SKOVE-3 (2), 1A9 (3), A2780 (4), CAOV-3 (5), OV-90 (6), and IGROV-1 (7) cells compared to HOSEpiC. As seen above, *Spry1* and *Spry2* were differently expressed in the cancerous cells. While Spry1 mRNA was almost undetectable in CAOV-3 cells, it was expressed by other cells, being more prominent in 1A9. Spry2 mRNA was identified in all cell lines with more prominent expression in cancerous ones. The product size for *Spry1* and *Spry2* was 438 bases and 471 bases, respectively. *β*-actin was used as a reference.

To evaluate the expression of Spry at mRNA level and its possible correspondence with Spry expression at protein level, we carried out reverse transcription polymerase chain reaction (RT-PCR) analysis on the total RNA samples derived from the normal and cancer cell lines studied. The expected product size for *Spry1* and *Spry2* was 438 bases and 471 bases, respectively. As shown in Figure 2, cancer cells showed variable levels of *Spry1*. While almost undetectable in CAOV-3 cells, *Spry1* was expressed by other cells, more remarkably by 1A9, suggesting that *Spry1* is differentially expressed in human epithelial ovarian cancer cells in vitro. *Spry2* was easily identified in all cell lines, with cancer cells expressing it in a more prominent way. 

### 3.4. Immunocytochemical Localization of Spry1 and Spry2 in OVCAR-3, SKOV-3, and HOSEpiC Cells

**Figure 3:**
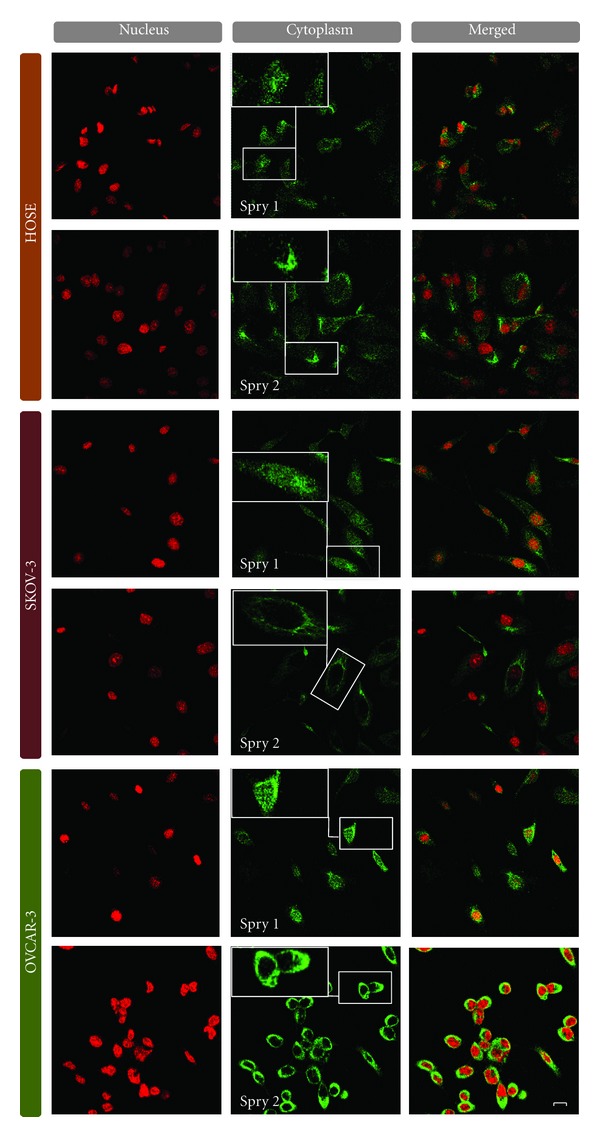
The subcellular localization of Spry1 and Spry2 proteins in primary human ovarian surface epithelial cell line HOSEpiC and human epithelial ovarian cancer cell lines OVCAR-3 and SKOV-3 using confocal immunofluorescence microscopy (60X oil immersion lense). Affinity-purified antibodies were used to specifically detect Spry1 and Spry2 (green). Propidium iodide staining was performed for nuclei (red). Two top rows represent HOSEpiC cells, two middle rows show SKOV-3 cells, and the bottom rows indicate OVCAR-3 cells. As seen, the staining intensity of Spry1 and Spry2 proteins was stronger in OVCAR-3 cells compared with HOSEpiC and SKOV-3 cells. Moreover, whereas Spry2 was predominantly distributed within the whole cytoplasm in vesicular structures, Spry1 was found in both cytoplasm and nucleus. Scale bar: 50 *μ*m.

To determine subcellular distribution of Spry1 and Spry2 proteins in human epithelial ovarian cancer, we performed confocal immunofluorescence microscopy on ovarian cancer cell lines OVCAR-3 and SKOV-3, as well as human ovarian surface epithelial cell line HOSEpiC, using antibodies specific for Spry1 and Spry2. These two cancer cell lines were selected based on the fact that they represent the cells with the highest and lowest expression of Spry1 and Spry2 amongst the cell lines studied, and that both are originally derived from ovarian serous adenocarcinoma, the most common subtype of epithelial ovarian cancer [18, 19]. The staining intensity of Spry1 and Spry2 proteins was stronger in OVCAR-3 cells compared with HOSEpiC and SKOV-3 cells. Moreover, whereas Spry1 was found in both cytoplasm and nucleus in vesicular structures, Spry2 predominantly showed a cytoplasmic localization (Figure 3).

### 3.5. Immunohistochemical Localization of Spry1 and Spry2 in Tumor Samples from Patients with Epithelial Ovarian Cancer Confirmed the Subcellular Distribution Pattern of the Protein Observed by Immunocytochemistry In Vitro

**Figure 4:**
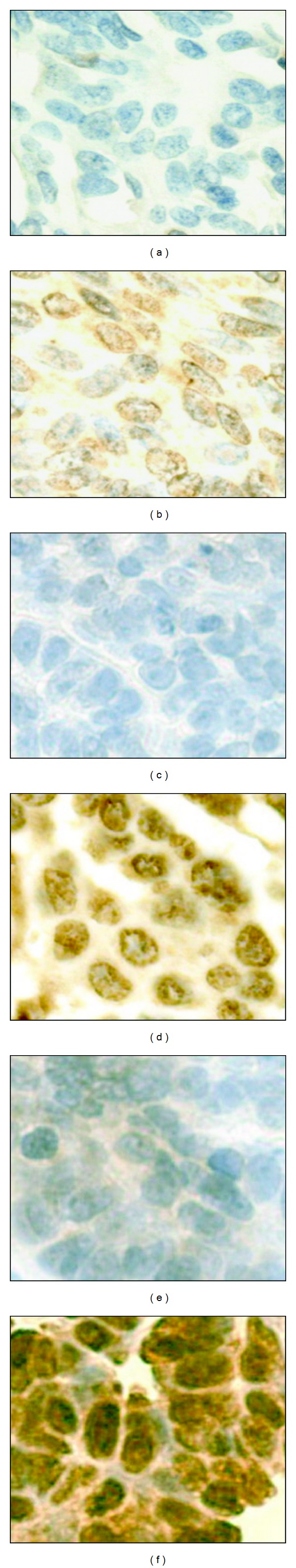
Immunohistochemical staining of Spry1 and Spry2 in paraffin-embedded slides from three different patients with epithelial ovarian cancer observed by Leica DMLB microscope (magnification ×40) and photographed using Leica DC200 digital imaging system. The blue color represents unstained parts and the yellow-brown color shows stained Spry with different intensity. As seen, Spry2 is expressed in cytoplasm (a), (c), and (e), while Spry1 staining is observed in both cytoplasm and nucleus (b), (d), and (f).

Using Spry1 and Spry2 specific antibodies, immunohistochemical staining was performed on random paraffin-embedded sections obtained from different patients. As seen in Figure 4, we observed a distribution pattern similar to what earlier revealed by our immunocytochemistry study, confirming a cytoplasmic and nuclear localization for Spry1 as well as a cytoplasmic distribution for Spry2.

## 4. Discussion

Based on prior reports on the role of Spry protein family in physiological and pathological conditions including human malignancies, we anticipated that the expression of one or more members of this protein family would be altered in epithelial ovarian cancer cell lines compared with normal ovarian epithelial cells. Examining the expression of Spry 1 and Spry2 in normal cells, our study indicates that surface epithelial cells of normal human ovaries express the two isoforms, in vitro. Haimov-Kochman et al. earlier reported the expression of Spry2 mRNA and protein in granulosa-lutein cells (GLC) of normal human ovaries [20]. These findings together could imply a role for Spry in ovarian physiology. Our results also indicate the differential expression of Spry1 and/or Spry2 across the ovarian cancer cell lines studied. These are, at least in part, in keeping with results from previous studies on other malignancies. Using immunohistochemical analysis and tissue microarrays, Kwabi-Addo et al. consistently showed downregulation of Spry1 protein in approximately 40% of prostate cancers compared with matched normal prostate [21]. Fong et al. also observed a consistent reduction in the expression of the Spry2 protein in malignant hepatocytes of human hepatocellular carcinoma (HCC) compared with normal or cirrhotic hepatocytes [8]. Downregulation of Spry2 expression in HCC was also confirmed in another study by Song et al. [22]. Sutterluty et al. reported a consistently decreased expression of Spry2 protein in nonsmall cell lung cancer (NSCLC) tissue and cell lines when compared with the normal lung epithelium [11]. Reduced expression of Spry2 protein was also reported in HT cell line which is derived from a human B-cell diffuse lymphoma [23]. Barbáchano et al. showed low levels of Spry2 in low-grade, but not high-grade, colorectal tumors [24]. In another study by Feng et al., reduced expression of Spry2 was observed more in patients with stage III or IV colon cancer than those with stage II disease, suggesting that downregulation of Spry2 in colon cancer may be associated with tumor invasion and metastasis [10]. Velasco et al. reported a reduction in spry2 expression in 19.85% of endometrial carcinoma and observed a strong and inverse correlation between Spry2 and cell proliferation. Considering the decreased expression of Spry2 in high grade tumors in comparison with low-grade carcinomas, they suggested that Spry2 could act as a “tumor progression” suppressor gene [25]. In the present study, it is not surprising that the pattern of alteration in Spry expression is not similar across the whole range of ovarian cancer cell lines studied. These cell lines are a number of commonly used in vitro representatives of various subtypes of epithelial ovarian cancer which are originally derived from individual patients demonstrating divergent clinicopathological characteristics. Moreover, all epithelial malignancies have a variety of genetic and epigenetic alterations and, in general, only a fraction of cases of a given tumor type has a specific alteration [21]. It should be noted in the present study that the expression of both Spry1 and Spry2 isoforms in OVCAR-3 cells was significantly higher than that in the other cancer cell lines and the control group, implying that although decreased expression of Spry1 and/or Spry2 was observed more frequently amongst the ovarian cancer cell lines studied, decline in the Spry expression might not necessarily be required in all epithelial ovarian cancers. 

A number of studies have indicated a diminished expression of some of Spry genes in breast [4], prostate [5–7], HCC [8, 9], NSCLC [11, 12], human B-cell lymphoma [26], and colon cancer [10]. However, Spry upregulation has been reported in melanoma cells carrying the B-Raf V599E mutation [13, 14], human patient-derived fibrosarcoma cell lines [27], and gastrointestinal stromal tumors (GIST) [15]. In a study by Schaner et al., the gene expression patterns of serous ovarian cancer tissue were investigated by cluster analysis [28]. The Spry homologs 1 and 2 were among the genes that demonstrated coexpression with some other specific genes. When compared to the western blot analysis, our evaluation of Spry presence at mRNA level indicates that Spry mRNA level may not necessarily correspond to the protein expression level. This might be due to the regulation of Spry protein family on various levels. This includes not only transcriptional and translational regulation, but post-translational modification resulting from protein differential localization and/or protein level modification via interaction with other proteins and mediators [29, 30]. Meanwhile, the expression of Spry at protein or mRNA levels seems to be cancer cell-type dependent, as well. In other words, alteration in the expression of Spry is not limited to its downregulation since increased expression of Spry has also been reported in some types of cancer. This scenario could be concluded by two different groups of studies. The first group suggests that some members of this protein family, in particular Spry2, may have a tumor suppressing potential. This is based on the observations showing Spry downregulation in different cancers (as described before), as well as the investigations demonstrating inhibitory effect of Spry family on ERK signaling pathway in Spry overexpressing cancer cells [8, 12, 21, 31]. The second group, on the other hand, suggests that while Spry may serve as an indicator of good clinical prognosis in cancers showing downregulation of Spry [32, 33], it is surprisingly a marker for poor clinical prognosis in other cancers with Spry upregulation [34]. This group is shedding light on the possibility of the later application of Spry proteins as tumor markers. Both aspects of Spry function appear to warrant further in-depth investigations. 

The localization of a protein can provide clues to its mode of action and/or function [35]. In the present study, we observed cytoplasmic and nuclear localization of Spry1 and only cytoplasmic localization of Spry2 in vitro. This was further confirmed by our immunohistochemical analysis of tumor samples from patients with ovarian cancer. Our findings concerning Spry2 localization were consistent with the results of studies by Hausott et al. [36] and Velasco et al. [25] demonstrating a cytoplasmic, but not nuclear, staining for Spry2 in NIH3T3 fibroblasts and human endometrial carcinoma samples, respectively. However, Hausott et al. revealed cytoplasmic and nuclear localization of Spry2 in DRG neurons and C6 glioblastoma cells [36]. To date, various cellular sites at which this protein family resides have been reported [35, 37] and the reasons for these discrepancies in Spry localization patterns are yet to be scrutinized.

## 5. Conclusion

In this study, we have addressed for the first time some aspects of Spry protein expression in human epithelial ovarian cancer. Our investigation unveiled the differential expression of Spry1 and Spry2 proteins in a range of human epithelial ovarian cancer cell lines. Our findings showing lack of correspondence between the expression of Spry mRNA and Spry protein favor the fact that Spry regulation occurs at various points. Also, our results showing the difference in subcellular localization between Spry1 and Spry2 isoforms might be of functional significance in ovarian cancer. The present report, along with similar studies on other cancers, suggests that the expression patterns of Spry protein family is cell-type dependent. Further studies exploring the effect of alterations in the expression of Spry on tumor cell biology are warranted. This could lead to the development of novel therapeutic strategies and reliable tumor markers for ovarian cancer. 

## Abbreviations

Spry: Sprouty
RT-PCR: Reverse transcription polymerase chain reaction
RTKs: Receptor tyrosine kinases
dSpry: *Drosophila melanogaster* Sprouty
EST: Expressed Sequence Tag database
GLC: Granulosa-lutein cells
HCC: Hepatocellular carcinoma
NSCLC: Nonsmall cell lung cancer
GIST: Gastrointestinal stromal tumors
ATCC: American Type Culture Collection
PBS: Phosphate-buffered saline
BSA: Bovine serum albumin.
