# Correction to “Initial Report on Differential Expression of Sprouty Proteins 1 and 2 in Human Epithelial Ovarian Cancer Cell Lines”

**DOI:** 10.1155/jo/9813231

**Published:** 2026-07-29

S. M. Moghaddam, A. Amini, A.‐Q. Wei, M. H. Pourgholami, and D. L. Morris, “Initial Report on Differential Expression of Sprouty Proteins 1 and 2 in Human Epithelial Ovarian Cancer Cell Lines,” *Journal of Oncology*, vol. 2012 (2012), https://doi.org/10.1155/2012/373826.

In the article titled “Initial Report on Differential Expression of Sprouty Proteins 1 and 2 in Human Epithelial Ovarian Cancer Cell Lines,” there was an error in Figure 1a. Specifically, the Western Blot bands representing the expression of GAPDH in the fourth panel were incorrectly oriented during figure assembly and Figure 1a should be corrected as follows:

**FIGURE 1:**
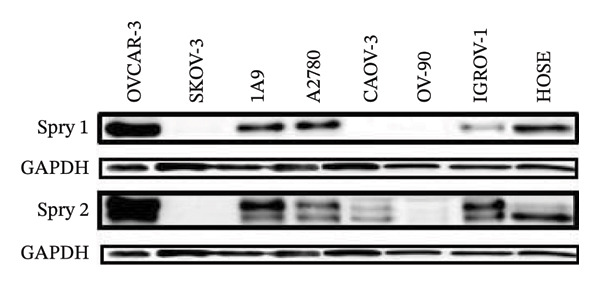
Expression of Spry1 and Spry2 proteins in a range of ovarian cancer cell lines compared with human ovarian surface epithelial cell line (HOSEpiC). (a) Western blot analysis of Spry1 and Spry2 expression in HOSEpiC, OVCAR‐3, SKOV‐3, 1A9,A2780, CAOV‐3, OV‐90, and IGROV‐1 cell lines using GAPDH as a loading control. The GAPDH loading control bands have been reused for the Spry1 and Spry2 analysis.

The authors would also like to add the following explanation to help the reader’s interpretation of the experimental process:

Sprouty‐1 and Sprouty‐2 migrate at approximately 34–36 kDa, while GAPDH migrates at approximately 36–37 kDa. In the experiments underlying Figure 1a, proteins were resolved on 10–12% SDS‐PAGE gels, which allow separation of proteins within the 30–40 kDa range, including differences of 1–3 kDa. Although these proteins are within a similar molecular weight range, they are distinguishable under these electrophoretic conditions.

For the blot presented in Figure 1a, a single PVDF membrane was used and sequentially probed. After detection of the first target protein, the membrane was stripped and reprobed to detect the subsequent targets, including GAPDH as the loading control. Detection was performed using mouse monoclonal antibodies for Sprouty‐1 and Sprouty‐2 (Abnova) and a monoclonal antibody for GAPDH (Sigma‐Aldrich), providing specificity for the expected bands.

Molecular weight markers were used during electrophoresis at the time of the experiment; however, as was common practice in many publications, the final figure presented cropped blot panels and did not include the marker lanes. Identification of each band was based on expected migration position, antibody specificity, and reproducibility across independent experimental replicates conducted at the time of the study.

We apologize for this error.

